# Blood flow restriction‐enhanced platelet‐rich plasma: A pilot randomised controlled trial protocol

**DOI:** 10.1002/jeo2.70034

**Published:** 2025-01-16

**Authors:** Muhammad Ayyan, Sushanth Alladaboina, Ayyoub Al‐Dolaymi, Mathieu Boudier‐Revéret, Emmanouil Papakostas, Theodorakys Marín Fermín

**Affiliations:** ^1^ Weill Cornell Medicine—Qatar, Qatar Foundation—Education City Doha Qatar; ^2^ Department of Surgery Aspetar Orthopaedic and Sports Medicine Hospital Doha Qatar; ^3^ Department of Physical Medicine and Rehabilitation University of Montreal Health Center Montreal Quebec Canada; ^4^ Centro Médico Profesional Las Mercedes, Av. Principal de Las Mercedes Caracas Venezuela

**Keywords:** blood flow restriction, injections, insulin‐like growth factor 1, interleukin 6, platelet‐rich plasma

## Abstract

**Purpose:**

To assess platelet‐rich plasma (PRP) changes in platelet and leucocyte count, insulin‐like growth factor 1 (IGF‐1), and interleukin 6 (IL‐6) concentration after bilateral low‐load knee extensions under blood flow restriction (BFR).

**Methods:**

The present randomised controlled trial protocol will include two groups: the intervention group, which will undergo bilateral knee extensions under BFR, and the control group, which will perform bilateral knee extensions without BFR. Participants will be randomly allocated in a 1:1 ratio. Twenty‐two healthy individuals will be enrolled if the predefined inclusion criteria are met: (1) males, (2) ages 18–40, (3) Tegner activity level ≥5 and (4) with no musculoskeletal conditions that would interfere with exercise. Exclusion criteria include (1) individuals with systemic inflammatory diseases, (2) cardiovascular risk factors, (3) any blood dyscrasia, (4) Tegner Activity scale scores <5, (5) under nonsteroidal anti‐inflammatory drugs and aspirin treatment within one week before testing or (6) that had previously performed exercises on the testing day. The participant will perform low‐load bilateral knee extensions under BFR following a standard protocol of 30‐15‐15‐15 repetitions of consecutive sets with 30‐s rest intervals at 80% of limb occlusive pressure and 30% of 1‐RM load. PRP platelet and leucocyte count, IGF‐1 and IL‐6 concentration measurements (via flow cytometry, chemiluminescence testing and immunochromatography, respectively) will be conducted before exercise and 10, 20 and 30 min after the intervention.

**Results:**

The expected outcome is that the standard protocol of low‐load bilateral knee extensions under BFR will increase the platelet and leucocyte count, IGF‐1 and IL‐6 in the PRP preparation.

**Conclusion:**

The current protocol allows the study of an enhanced PRP formulation for its potential implementation in multiple sports injuries.

**Abbreviations**
1‐RM: one‐repetition maximum
BFR: blood flow restriction
IGF‐1: insulin‐like growth factor 1
IL‐6: interleukin 6
PRP: platelet‐rich plasma

## BACKGROUND

Platelet‐rich plasma (PRP) is a portion of autologous blood with increased platelet concentration obtained by centrifugation [43]. This orthobiologic therapy has been increasingly implemented during the last two decades, especially in knee osteoarthritis and tendinopathies, with promising outcomes [14, 16, 38]. Although the wide variety of available PRP products and the poor reporting quality of the PRP preparations have limited its standardisation, platelet and leucocyte doses have been established as key factors in this orthobiologic therapy [7, 36, 37].

Among PRP components, insulin‐like growth factor 1 (IGF‐1) and interleukin 6 (IL‐6) have critical roles. IGF‐1 has been associated with the proliferation and maturation of chondrocytes and inhibiting the apoptosis of osteoarthritic chondrocytes (via PDCD5 downregulation) [27, 42, 51]. On the other hand, IL‐6 is a proinflammatory cytokine that has been linked with cartilage matrix degradation (via metalloproteinases) [2] but potentially promotes muscle growth by activating satellite cells to repair damaged muscle fibres [8] and neovascularization [46] and induces tendon repair by collagen production [23, 35, 47, 48].

Blood flow restriction (BFR) training is a rehabilitation modality with local and systemic effects on the musculoskeletal system [44] that has been associated with the release of IGF‐1 [31, 40, 49] and IL‐6 [9, 15, 31, 39, 45] in the bloodstream. This training modality consists of the application of a tourniquet in the proximal area of the upper or lower limb with partial occlusive pressures (from 40% to 80% of the limb occlusive pressure) to create a venous stasis and promote a metabolic stress environment that triggers the release of multiple hormones and growth factors with local and remote effects [4, 5, 6, 21, 26].

BFR training allows the implementation of loads as low as 20%–30% of the one‐repetition maximum (1‐RM) (the weight load that would allow performing only one successful repetition of a specific exercise) compared to the 70% of the 1‐RM of the traditional resistance training [6, 26]. The BFR standard protocol comprises a 30‐15‐15‐15 repetition of consecutive sets with 30‐s rest intervals for a total of 75 repetitions with 20%–30% of the 1‐RM load [6, 26].

The combination of the tourniquets with low‐load training results in similar muscle strength and hypertrophy gains as the traditional weight resistance training with high loads, attracting much interest for postoperative knee rehabilitation [3, 50]. The growth hormone mediates this muscle hypertrophy [29], IGF‐1 and myokines, specifically, IL‐6 (promoting muscle regeneration and increased protein synthesis), driven by anaerobic glycolytic metabolism, glycogen depletion and intramuscular hypoxia [52]. A significant increase in these factors has been reported as early as in the first 30 min postexercise and as high as 290 times for growth hormone [9, 31, 49].

Different exercise modalities consistently alter plasma composition [1, 24, 53] and increment platelet concentration by over 20% [18] (Table 1). Analysing PRP composition after BFR training supposes a potentially enhanced PRP formulation with increased platelet count, IGF‐1 and IL‐6 levels that could benefit patients with cartilage, tendon and muscle pathologies (Table 2). BFR can be performed safely [22] and at a low cost in an office setting and easily implemented before blood draws for PRP preparation, resulting in an easy and fast intervention to improve PRP composition for treating sports‐related injuries.

**Table 1:** Exercise modalities and their effects on plasma and PRP composition.

References	No. of patients	Exercise modality	Result
Hamilton et al. [16]	10	Modified submaximal cycling test on an electronically braked cycle ergometer at 50% of peak power output for 1 h.	Increased platelet, leucocyte and PDGF‐AB in PRP. No effect on IGF‐1 and hepatocyte growth factor.
Baria et al. [6]	10	After a 5‐min warm‐up, the participants engaged in interval exercise using an exercise bike, completing eight intervals. Each interval consisted of 20 s of work followed by 10 s of rest at strenuous or maximum intensity according to Borg's rate of perceived exertion.	Increased platelet count and TGF‐β levels in PRP. The other cellular components (leucocytes, red blood cells and mean platelet volume) and growth factors (PDGF, IGF‐1 and VEGF) were not significantly changed.
Callanan et al. [7]	16	Testing was performed on the recumbent cross‐trainer wearing a cooling vest set at a temperature of 8.3°C. Blood flow restriction cuffs were applied bilaterally on the upper arm and upper leg at 40‐ and 65‐mm Hg of compression throughout exercise testing. The participants sat on a cooling pad at 8.3°C (47°F). Testing was performed with the participant barefoot because the machine's foot panels also provided cooling. The participants were placed on the machine for 20 min of exercise. Once the warm‐up was completed, the participants performed six sprint intervals, alternating 30 and 60‐s sprints at maximal exertion effort. Following the 30‐s sprints, the participants had 1.5 min of active recovery before the 60‐s sprint. Two minutes of active recovery followed each 60‐s sprint.	Increased leucocyte count is marked by an increase in lymphocyte differential and a decrease in neutrophil differential in blood samples. Increased platelet count and CD34+ cells but decreased monocyte count. No differences in IL‐10, IL‐6, granulocyte‐macrophage colony‐stimulating factor, IL‐1ra, TNF‐α or IL‐2 were observed.
Anz et al. [4]	20	Participants followed an exercise regimen on an upright bike. The exercise regimen involved a 5‐min warm‐up period followed by 20 min of vigorous exercise, which was determined by maintaining the target heart rate at 70% to 85% of the maximum target heart rate.	Increased platelet count (by over 20%), leucocytes and hematopoietic progenitor cells in PRP.

Abbreviations: IGF‐1, insulin‐like growth factor 1; IL, interleukin; PDGF, platelet‐derived growth factor; PRP, platelet‐rich plasma; TGF‐β, transforming growth factor beta; TNF‐α, tumour necrosis factor alpha; VEGF, vascular endothelial growth factor.

**Table 2:** Clinical trials on local and systemic effects of BFR.

References	No. of participants	BFR protocol	Outcome measures	Result
Takarada et al. [51]	6	Exercise: bilateral knee extension exercise (0°–90°) in a seated position with an isotonic leg extension machine. Sets, repetitions and rest periods: five sets until failure at a ∼1‐s constant velocity and 30‐s rest intervals. Duration: single session. Load: ∼20% 1‐RM. Cuff placement: proximal thigh. Occlusive pressure: the mean pressure given by the tourniquet was 214 ± 7.7 (SE) mmHg. The exercise session lasted for ∼10 min, and the pressure was released immediately after the exercise session.	Electromyogram. Plasma concentrations of growth hormone, norepinephrine, creatine phosphokinase, IL‐6 and lipid peroxide. Five minutes before exercise, immediately after exercise, and 15, 45, 90 min, and 24 h after exercise.	Low‐load resistance exercise combined with BFR causes enhanced muscular electrical activity and plasmatic increased growth hormone concentrations with no serious muscle tissue damage. However, a slight elevation of plasma IL‐6 concentration suggests finer microdamage within vascular walls and/or muscle tissue.
Takano et al. [50]	11	Exercise: bilateral leg extension exercise with the lower extremity at approximately 90° flexion. Sets, repetitions and rest periods: 30 repetitions without rest, and after a 20‐s rest, they performed three sets again until exhaustion. Duration: two sessions separated by 2–4 weeks. Load: 20% of 1‐RM. Cuff placement: both legs were pressure‐applied by a specially designed belt named Kaatsu in Japanese. Occlusive pressure: under the conditions with Kaatsu, the arterial flow was reduced to about 30% of the control.	The superficial femoral arterial blood flow and hemodynamic parameters were measured using ultrasound and impedance cardiography. Serum concentrations of growth hormone, vascular endothelial growth factor, noradrenaline, IGF‐1, ghrelin and lactate were also measured 0 ~ 1, 10 and 30 min after exercise.	Short‐term low‐intensity resistance exercise with Kaatsu significantly increased growth hormone, IGF‐1 and vascular endothelial growth factor. The increase in growth hormone was related to neither norepinephrine nor lactate, but the increase in vascular endothelial growth factor was related to that in lactate. Ghrelin did not change during the exercise. The maximal heart rate and blood pressure in short‐term low‐intensity resistance exercise with Kaatsu were higher than without Kaatsu. Stroke volume was lower due to the decrease of the venous return by Kaatsu, but total peripheral resistance did not change significantly.
Reeves et al. [43]	8	Exercise: light resistance BFR (a) single arm biceps curls with a dumbbell; (b) calf extensions; (c) no loads) and moderate resistance (traditional exercise with 70% RM and no BFR). Sets, repetitions and rest periods: three sets of full range‐of‐motion contractions to failure, following the cadence of an audible metronome set to 0.67 Hz, with 1‐min rest intervals. Duration: three single sessions on different days. Load: 30% 1 RM. Cuff placement: (a) above the biceps brachii; (b) above the gastrocnemius; (c) above the biceps brachii. Occlusive pressure: 20 mmHg below the acute systolic pressure, with maintenance of cuff occlusion throughout and for 1 min following completion of the final set.	Plasmatic lactate, growth hormone, testosterone, free testosterone and cortisol levels. Blood samples were taken pre‐, postexercise, and 15 min postexercise for each experimental condition.	Similar lactate responses were obtained between the light resistance partial occlusion and moderate resistance trials, indicating the same metabolic stress produced by both trials. However, light resistance partial occlusion elicited a greater growth hormone response than moderate resistance, but testosterone, free testosterone and cortisol were not affected.
Patterson et al. [38]	10	Exercise: single‐leg plantar‐flexion resistance exercise. Sets, repetitions and rest periods: three sets of exercise to the point of failure, at a cadence of 1.5 s lifting and 1.5 s lowering the weight, with a 1‐min rest interval between sets. Duration: three weekly sessions for 4 weeks. Load: 25% 1‐RM. Cuff placement: proximal calf. Occlusive pressure: 110 mmHg was maintained for the three sets (including rest periods), resulting in a restriction period of ~5–8 min.	Muscle strength: 1‐RM, maximal voluntary contraction, isokinetic torque at baseline and 4 weeks. Limb blood flow: postocclusive blood flow at baseline and 4 weeks.	Low‐load resistance training with BFR improved maximal strength (1‐RM, maximal voluntary contraction, isokinetic torque at 0.52 rad/s) and peak postocclusive blood flow compared to low‐load resistance training with normal blood flow.
Manini et al. [27]	20 (10 older and 10 young men)	Exercise: bilateral knee extensions. Sets, repetitions and rest periods: four sets until volitional fatigue, with 2‐min rest intervals between sets. Duration: two exercise sessions separated by a minimum of 4 days. The average number of days between testing sessions was 13 days. Load: (a) 80% 1‐RM without BFR (high‐load resistance exercise) and (b) 20% 1‐RM with concurrent BFR. Cuff placement: upper thigh. Occlusive pressure: 1.5 times each subject's brachial systolic blood pressure (range: 135–186 mmHg) and initiated 2 min before exercise. The cuff remained inflated for the duration of the exercise and rest periods.	Plasmatic levels of growth hormone, IGF‐1 and lactate at 10‐min intervals beginning 30 min prior to exercise and concluding 120 min after the onset of exercise. Exercise volume, perceived exertion and pain.	Older men exhibited a blunted maximal growth hormone response to low‐load resistance exercises with BFR compared to younger men, and young men experienced a higher maximal growth hormone concentration but similar area under the curve growth hormone concentrations following low‐load resistance exercises with BFR despite performing a lower total exercise volume with BFR. In addition, circulating IGF‐I was substantially lower in older men and remained unaltered by either exercise protocol.
Suga et al. [49]	12	Exercise: ankle plantar flexion exercises in four conditions. Two resistance exercises without BFR and two BFR exercise protocols. Sets, repetitions and rest periods: three sets of 1‐min exercise with 30 repetitions per set, lifting the weight 5 cm above the ground with 1‐min rest intervals between sets. Duration: each experimental exercise session lasted 5 min. Subjects were tested on two occasions, separated by at least 48 h. On each experimental day, the interval between conditions was at least 30 min. Load: the two resistance exercises without BFR were low‐intensity and high‐intensity, at 20 and 65% 1‐RM, respectively. Cuff placement: right thigh. Occlusive pressure: 130% of the subject's resting systolic blood pressure and 144 ± 21 mmHg on average. The two BFR protocols were performed with intermittent or continuous BFR combined with low‐intensity exercises. In the intermittent BFR protocol, the BFR was applied only during exercise. The pressure cuff was inflated before every set and released after the finish of sets. In the continuous BFR protocol, the BFR was maintained throughout the 5‐min exercise session.	Intramuscular metabolic stress, defined as intramuscular metabolites and pH, and muscle fibre recruitment were evaluated by P‐magnetic resonance spectroscopy at rest and during exercise.	The changes in intramuscular metabolites and pH during intermittent BFR were significantly greater than those in low‐intensity exercises but significantly lower than those in high‐intensity exercises. By contrast, those changes in continuous BFR were similar to those in high‐intensity exercises. Moreover, the fast‐twitch fibre recruitment, evaluated by a splitting Pi peak, showed a similar level to high‐intensity exercises.
Patterson et al. [39]	7	Exercise: unilateral knee extension exercise. Sets, repetitions and rest periods: five sets until failure with 30 s rest between each set. Immediately after the fifth set, participants repeated the exercise with the contralateral leg. Duration: participants completed the two trials in a counterbalanced order, lasting 7–10 days between trials. Load: 20% 1‐RM. Cuff placement: upper thigh. Occlusive pressure: 110 mmHg, maintained throughout the exercise (8–10 min), including rest periods.	Plasma concentrations of growth hormone, IGF‐1, vascular endothelial growth factor, cortisol and IL‐6 at rest and 30‐, 60‐ and 120 min postexercise.	A single bout of low‐load resistance training with BFR increases the circulating concentrations of growth hormone and vascular endothelial growth factor in older men. It may explain the skeletal muscle and peripheral vascular adaptations observed following training with BFR.
Nielsen et al. [36]	This study consisted of two distinct intervention protocols (3‐week study and 1‐week study) using two separate study populations for a total of 36 participants.	Exercise: (a) low‐load unilateral knee extension exercise with BFR; (b) low‐load unilateral knee extension exercise without BFR and (c) high‐load unilateral knee extension exercise without BFR. Sets, repetitions and rest periods: four sets of unilateral knee extension exercise until concentric failure at a concentric/eccentric phase cadence of 1.5 s/1.5 s with 30 s and 90 s for low‐load and high‐load exercises, respectively. Duration: (a) 3‐week training programme consisting of 23 exercise sessions, 1–2 daily training sessions from Monday to Friday, separated by at least 4 h. b) 1‐week supervised training programme involving seven or three exercise sessions for BFR and high‐load exercises. BFR exercise performed high‐frequency exercise once per day (Monday, Tuesday, Friday) and twice per day (Wednesday, Thursday) while high‐load exercise trained once per day (Monday, Wednesday, Friday). Successive training sessions were separated by at least 4 h. Load: 20% for BFR training and low‐load exercises without BFR and 70% 1‐RM for high‐load training without BFR. Cuff placement: upper thigh. Occlusive pressure: 100 mmHg.	Macrophage (M1/M2) content, heat shock protein (HSP27/70) and tenascin‐C expression in muscle biopsies obtained at baseline, 8 days into the intervention and 3 and 10 days after training cessation. Markers of muscle damage (creatine kinase), oxidative stress (total antibody capacity, glutathione) and inflammation (monocyte chemotactic protein‐1, IL‐6, tumour necrosis factor α) in blood samples collected before and after (0.1–24 h) the first and last training session.	Myocellular infiltration of pro‐ and anti‐inflammatory (M1/M2) macrophages increased 3 days following 19 days of high‐frequency BFR training. A similar pattern emerged for work‐matched free‐flow training but involving proinflammatory macrophages only. Second, HSP27 expression was upregulated after 1 week of BFR training, while both HSP27 and HSP70 were upregulated in the early recovery phase after cessation of training in work‐matched controls. Third, low‐load BFR training and high‐load free‐flow strength training demonstrated highly similar changes in circulating markers of myocellular damage, inflammation and oxidative stress in response to acute and 1 week of exercise training and whenever within‐group increases emerged this was exclusively in response to high‐load free‐flow exercise.
Korakakis et al. [19]	30	Exercise: knee extensions. Sets, repetitions and rest periods: four sets of knee extensions; one set to maximum possible repetitions (2 s concentric, 2 s eccentric phase, paced by a metronome), then three sets of 15 repetitions with 30 s rest between sets. Termination of the first set was indicated either by the inability to follow the pace of the metronome or the inability to extend the knee joint fully. Duration: single session. Load: a maximum of 5 kg (variable ankle weights) such that the patient reported a maximum of 4/10 during familiarisation (without BFR inflation). Cuff placement: upper thigh at the inguinal region. Occlusive pressure: 80% of the limb occlusive pressure. The BFR cuff was kept inflated throughout the session.	Pain (0–10) was assessed immediately after BFR application and after a physiotherapy session (45 min) during shallow and deep single‐leg squats and step‐down tests.	Significant effects were found with greater pain relief immediately after BFR in shallow and deep single‐leg squats and step‐down tests. Time analysis revealed that pain reduction was sustained after the physiotherapy session for all tests. The decrease in pain effect size was clinically significant in both post‐BFR assessments.
May et al. [30]	24	Exercise: the training programme comprised 20 resistance exercise sessions in which unilateral bicep curls were performed in the dominant arm only, followed by bilateral knee extension and bilateral knee flexion exercises in each training session. Sets, repetitions and rest periods: 30 repetitions, followed by three sets of 15 repetitions separated by 30‐s rest periods. All lifts were performed at a 2‐s eccentric and 2‐s concentric cadence, guided by a metronome. Duration: 4 sessions over 8 weeks. Load: 30% 1‐RM. Cuff placement: upper thighs. Occlusive pressure: 60% of the participants' limb occlusive pressure. The total time under restriction was roughly 14 min.	Strength: bilateral leg exercises and unilateral bicep curls 1‐RM were measured using trained and untrained arms. The muscle cross‐sectional area was measured via peripheral quantitative computed tomography of the dominant leg and both arms.	1‐RM in the trained arm increased more in the experimental group than the contralateral untrained and the trained arm of the control group. The increase in knee extension 1‐RM was twofold that of control. Knee flexion 1‐RM, leg cross‐sectional area, and trained arm cross‐sectional area increased similarly between groups, while untrained arm cross‐sectional area did not change.

Abbreviations: 1‐RM, one‐repetition maximum; BFR, blood flow restriction; IGF‐1, insulin‐like growth factor‐1; IL, interleukin.

The present study aims to assess PRP changes in platelet and leucocyte count, IGF‐1 and IL‐6 concentration after bilateral low‐load knee extensions under BFR. The hypothesis is that bilateral low‐load knee extensions under BFR will increase platelet and leucocyte count, IGF‐1 and IL‐6 in PRP prepared after the exercise bout.

## METHODS/DESIGN

### Study design

This study will be a randomised controlled trial. The study design will include two groups: the intervention group, which will receive the BFR during knee extensions, and the control group, which will perform knee extensions without BFR.

### Setting

The research will be conducted at two locations in Caracas, Venezuela. A private ISO:9001‐certified laboratory (Laboratorio Avilab) with over 40 years of experience in conducting multiple blood tests, immediate result delivery, and facilities capable of drawing blood from up to eight patients simultaneously; and the principal investigator private practice where his team regularly performs PRP injections and BFR training.

### Participants and eligibility criteria

The principal investigator will assess patients undergoing routine screening for a 3–4‐week period. A timeline of the study's phases is summarised in Table 3. Twenty‐two healthy individuals will be enrolled by the investigators if the predefined inclusion criteria are met: (1) males, (2) ages 18–40, (3) Tegner activity level ≥5 and (4) with no musculoskeletal conditions that would interfere with exercise. Exclusion criteria include (1) individuals with systemic inflammatory diseases, (2) cardiovascular risk factors (including deep vein thrombosis, hypertension, lymphedema, history of endothelial dysfunction, varicose veins, peripheral vascular disease), (3) any blood dyscrasia, (4) Tegner Activity scale scores <5, (5) under nonsteroidal anti‐inflammatory drugs and aspirin treatment within one week before testing or (6) that had previously performed exercises on the testing day.

**Table 3:** Timeline of the study's phases.

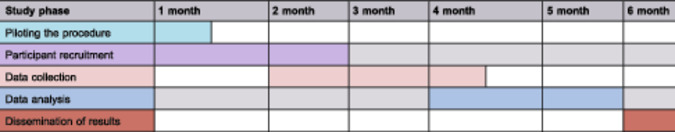

Eligible participants will be offered enrolment after discussing the research plan in detail, providing the patients with an informative leaflet and signing written informed consent. This leaflet contains detailed information about the study, including its purpose, procedures, potential risks and benefits, and a comprehensive description of how personal data will be handled. Participants will be allowed to withdraw from the study at any time.

### Handling of participant loss

Patients with missing data due to dropout during the study will be excluded, meaning only patients with a complete data set will be eligible for the final analysis.

### Randomisation and allocation

**Figure 1:**
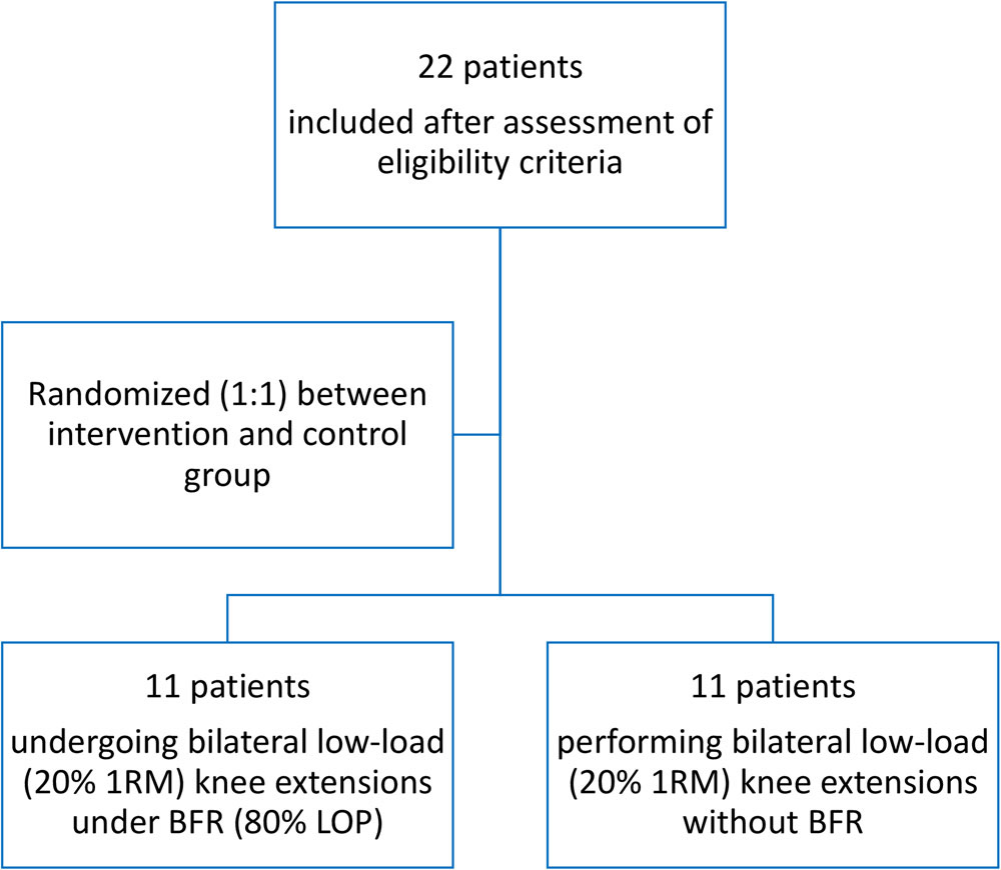
Participant recruitment, randomisation and group allocation flowchart. 1‐RM, one‐repetition maximum; BFR, blood flow restriction.

Participants will be randomly allocated into the intervention and control groups in a 1:1 ratio. The allocation sequence to either group will be generated using computer‐generated random numbers, ensuring that the assignment of participants to either the intervention group or the control group is entirely random and unbiased (Figure 1).

### Intervention and blood sample collection

A multidisciplinary medical team led by the principal investigator will conduct the intervention in an ISO:9001‐certified laboratory (Laboratorio Avilab) (Table 4). Before the intervention, participants will have a pre‐exercise peripheral vein catheterisation, blood sample draw and PRP preparation for baseline measurements. Each participant will undergo standard venipuncture in the antecubital fossa by a single phlebotomist under sterile conditions for a total blood draw of 15 mL in a vacutainer and undergo a single centrifugation at 1500 rpm for 5 min. The plasma portion and buffy coat will be separated under direct visualisation with automatic pipettes from the red blood cells, and samples will be sent to the laboratory and divided into two aliquots, one for cell counts (automated cell counter), the other for IGF‐1 and IL‐6 immunoassays analysis within 6 h.

**Table 4:** Summary of procedural steps during the test day.

**Procedural steps** Evaluation of eligibility in athletes undergoing routine screening, including Tegner Activity Scale assessment. Informed consent. Demographic data collection. Preblood flow restriction blood sample collection. Low‐load blood flow restriction bilateral knee extension intervention. Postblood flow restriction blood sample collection at 10', 20' and 30'. Laboratory testing of collected samples.

The participant will then perform the low‐load BFR protocol. The low‐load bilateral knee extensions under BFR (using tourniquets at the proximal end of both thighs) will follow the standard protocol of four sets consisting of 30‐15‐15‐15 repetitions, with 30‐s rest intervals at 80% of limb occlusive pressure (calculated using arteria pedis ultrasound) and 30% of 1‐RM load (using Holten diagram).

A staff physician will monitor the entire exercise protocol and recovery period for safety and adverse events. Once the exercise protocol is completed, participants will be allowed a recovery period (including rest, walking if desired, and fluid intake) before undergoing the consecutive blood draw. The maximum recovery permitted time will be 5 min. The subsequent blood draws and PRP processing will be performed identically to the first at 10‐, 20‐ and 30‐min postintervention. The average test participation will last around 2 h.

### Outcome assessment

Measurements will be taken before and 10, 20 and 30 min after the intervention. The primary outcomes of this study are directly related to the hypothesised benefits of BFR‐enhanced PRP. The expected result is that the standard protocol of low‐load bilateral knee extensions under BFR will increase the platelet and leucocyte count, IGF‐1 and IL‐6 in the PRP preparation. Increases in platelet and leucocyte counts and elevated IGF‐1 and IL‐6 concentrations within the first 30 min after the exercise bout could indicate enhanced healing and anti‐inflammatory effects that could be translated into an office setting, potentially leading to improved outcomes in musculoskeletal pathologies.

The study's data quality relies on standardised procedures and thorough research team training. This includes the utilisation of a single phlebotomist for all blood draws, the oversight of the principal investigator during the exercise protocol and two certified bioanalysts to individually handle IGF‐1 and IL‐6 measurements. The XS‐i Fluorescent Flow Cytometer (Sysmex) will be employed for leucocyte differential analysis, providing essential information on the various types of white blood cells. The XS‐i Automated Hematology Analyzer (Sysmex) will be used for electrical impedance analysis, measuring platelet, red cell and leucocyte counts. The Finecare FIA Meter Plus Analyzer (Wondfo) will conduct fluorescence immunochromatographic analysis of IL‐6, a significant biomarker in this research. The LIAISON Analyzer (DiaSorin) will also perform IGF‐1 chemiluminescence testing, which is crucial for measuring IGF‐1 levels. The implemented BFR System (The Occlusion Cuff Pro®) is specifically designed to apply BFR during the exercise protocol, ensuring accurate and consistent application of BFR across all participants.

### Benefits/risks

Patients will benefit from the systemic and local musculoskeletal effects of BFR training and receive their blood test results for annual control.

BFR training is safe, with risks consistent with traditional exercise modalities [22]. However, sparse reports of common side effects include pain or discomfort during exercise, delayed‐onset muscle soreness and cardiac stress (increased heart rate, increased blood pressure, decreased stroke volume). In contrast, more serious, less common side effects include numbness or nerve injury, bruising or ischaemic injury, dizziness or fainting, thrombus formation, muscle damage and rhabdomyolysis. These risks are negligible when BFR is performed under current standards.

Contraindications for BFR implementation include a history of or the potential for deep vein thrombosis, blood clotting disorder, poor circulation, hypertension, inadequate function of the lymphatic system, history of endothelial dysfunction, varicose veins, peripheral vascular disease, diabetes, easy bruising, active infection, cancer, renal compromise, pregnancy and intervention intolerance.

Potential adverse effects will be recorded in a logbook and promptly reported to the Research and Bioethics Committee. A follow‐up call will be done 72 h after the blood sample collection to assess for late presentation of any adverse effects.

### Data integrity

The medical record numbers will be paired with a unique research identification number and stored on a separate spreadsheet to protect against a breach of privacy. Data storage includes secure electronic storage with restricted access, regular data backups and compliance with data protection regulations. Also, the link between code and identifier will be destroyed after the study is finished, and de‐identified data will be kept for at least 5 years. The principal investigator will monitor the data collected, including those related to unanticipated problems and adverse events. Any complications that arise during the trial must be reported immediately. In the event of data breaches, these should be reported to the project coordinator within 24 h of the incident being identified, ensuring prompt action to mitigate potential risks.

### Statistical analyses

The study will involve a sample size of 22 patients. This sample size has been validated by a power analysis, which anticipates a 25% increase in IGF‐1 with a significance level (α) of 0.05 and 80% statistical power. Additionally, previous studies have been conducted using a similar sample size [18, 24, 53].

Patient data will be summarised using the following descriptive statistics: mean and standard deviation, median and interquartile range and/or frequency and percentage (%). The distribution of numerical variables will be assessed using the Shapiro–Wilk test. For numerical variables, the repeated‐measures analysis of variance test will be used for those with a normal distribution, and the Friedman test will be used for those with a nonnormal distribution. Values of *p* < 0.05 will be considered statistically significant. Statistical analysis will be performed using SPSS version 19 and Microsoft® Excel® version 2016.

## DISCUSSION

The present randomised controlled trial aims to assess PRP changes in platelet and leucocyte count, IGF‐1 and IL‐6 concentration after bilateral low‐load knee extensions under BFR. The muscle metabolic stress induced by BFR has been associated with releasing IGF‐1 and IL‐6 into the bloodstream [9, 15, 31, 39, 40, 45, 49]. It can be performed safely [22] and implemented at a low cost in an office setting, making it an easy and fast intervention to potentially enhance PRP composition for treating sports‐related injuries.

Evidence of different PRP compositions targeting specific musculoskeletal pathologies is growing [13, 20, 25, 30, 41]. Leucocyte‐rich PRP preparations have been reported to be better in tendinopathies, while leucocyte‐poor are preferred for knee osteoarthritis [13, 20, 25, 30, 34, 41, 54]. The potential effects of BFR represent an exciting opportunity to increase the concentration of IGF‐1 and IL‐6 in PRP, with a synergistic effect that can benefit specific injuries like muscle injuries (hamstrings) and other cartilage and tendon pathologies. Literature on BFR implementation in major sports injury rehabilitation, such as ACL reconstruction [17, 32], is rapidly growing, and several authors have expressed its potential in treating muscle injuries [19, 28, 33].

With a BFR‐enhanced PRP formulation displaying a higher concentration of any or both IGF‐1 and IL‐6, there is an opportunity for treating muscle injuries [8, 29, 46, 52]. Although both molecules have been shown to contribute to musculoskeletal tissue healing, the chance to treat muscle injuries is the most exciting, as the current evidence on PRP has shown poor outcomes [7, 10, 11, 12]. The protocol allows the study of an enhanced PRP formulation for its potential implementation in multiple sports injuries.

## AUTHOR CONTRIBUTIONS

**Muhammad Ayyan**: Resources; writing—original draft. **Sushanth Alladaboina**: Resources; writing—original draft. **Ayyoub Al‐Dolaymi**: Validation; supervision; funding acquisition. **Mathieu Boudier‐Revéret**: Methodology; data curation; writing—review and editing. **Emmanouil Papakostas**: Validation; supervision. **Theodorakys Marín Fermín**: Conceptualisation; methodology; formal analysis; investigation; resources; data curation; writing—review and editing; visualisation; project administration.

## CONFLICT OF INTEREST STATEMENT

The authors declare no conflicts of interest.

## ETHICS STATEMENT

Ethical approval has been received from the Research and Bioethics Committee of Grupo Médico Vargas—Clínica Santa Sofía v.001‐2024 in February 2024. The trial has been prospectively registered with the BioMed Central—International Standard Randomised Controlled Trial Number (ISRCTN42221463).

## Data Availability

The data underlying this article are available in the article and its online supplementary material.
